# Two Faces of a Coin? A Systematic Review of Source Monitoring and Its Relationship with Memory in Autism

**DOI:** 10.3390/brainsci11050640

**Published:** 2021-05-15

**Authors:** Stefano Damiani, Cecilia Guiot, Marta Nola, Alberto Donadeo, Nicola Bassetti, Natascia Brondino, Pierluigi Politi

**Affiliations:** Department of Brain and Behavioral Sciences, University of Pavia, via Bassi 21, 27100 Pavia (PV), Italy; stefano.damiani01@ateneopv.it (S.D.); marta.nola@unipv.it (M.N.); alberto.donadeo01@universitadipavia.it (A.D.); nicola.bassetti01@universitadipavia.it (N.B.); natascia.brondino@unipv.it (N.B.); pierluigi.politi@unipv.it (P.P.)

**Keywords:** autism, Asperger’s, source monitoring, source memory, reality monitoring, action monitoring, old–new recognition, recall, self–other distinction, enactment effect

## Abstract

The ability to discriminate the origin of stimuli, known as source monitoring, is crucial for self–other distinction and the integration of internally generated and externally generated experiences. Despite its valence, evidence on source monitoring in autism is yet scarce and unclear. We systematically reviewed literature concerning source monitoring in autism and its relationship with other constructs, such as memory type, encoding effects, social cognition, general intelligence, and clinical factors. Source-monitoring performance (operationalized as error or accuracy) was reduced in autistic participants in 9 of the 15 studies that met the inclusion criteria. When explicitly investigated, free-recall memory impairments in autism were shown to influence source monitoring deficits. General intelligence was another important factor linked to source-monitoring performance. Conversely, other memory types or encoding effects were not impaired in autism, and no univocal association could be found with source monitoring. Social cognition and clinical symptoms were rarely assessed in spite of their possible involvement in source monitoring. The heterogeneity of the task design, outcome measures and demographical factors limited study comparability. As a research framework on source monitoring as a construct of primary interest in autism is still lacking, we propose preliminary indications for future investigations based on the collected findings.

## 1. Introduction

Autism spectrum disorder (ASD) is a neurodevelopmental condition diagnosed through the identification of anomalies in social communication, restrictive interests, and repetitive patterns of behavior [1]. Autistic individuals often display atypical processing of both self-related and other-related information [2]. Several lines of research raised the question of whether ASD involves a decreased ability to use the self as an effective organizational strategy to encode stimuli and experiences [3], showing alterations in self–other distinction and integration [4,5,6].

In this context, the investigation of source monitoring (SM) in ASD has gained relevance. As defined by Mitchell [7], SM is the ability to discriminate or remember the origin of a specific stimulus. Through this mental activity, a subject can make attributions about the source of subjective experiences, distinguishing, for example, between a perception, a memory, a belief, or something that has been only imagined. The most widespread framework for SM differentiates (i) reality monitoring (RM), whereby the subject distinguishes external and internal sources (for example, “did I listen to another person reading a word, or was it me who pronounced it?”); (ii) internal source monitoring (ISM) —discriminating between internal sources (“did I think of it or did I say it aloud?”) and (iii) external source monitoring (ESM) —discriminating between external sources (“did John say it, or was it said on TV?”) [8].

Errors in SM have been largely studied not only in neurotypical individuals, but also in neurological and psychiatric conditions, such as Alzheimer’s [9] and schizophrenia [10]. Conversely, studies in ASD have been less numerous and systematic, and evidence for SM abnormalities in ASD has been conflicting so far. This is not surprising considering that SM performance may be greatly influenced by other cognitive resources that are also impacted in ASD. Above all these functions, the role of memory is fundamental when evaluating SM: on one hand, most SM tasks also involve recall and recognition memory testing [11]. On the other, only specific memory types are impaired in ASD [12]. Furthermore, memory is modulated by encoding effects, which have been hypothesized to operate differently in ASD [13]. Encoding effects are preceded by self–other differentiation, and thus take place after source discrimination processes. For example, the enactment effect refers to enhanced memory for self-performed compared to other-performed actions [14], while the observer effect describes the opposite phenomenon [13]. Finally, the intention superiority effect poses that individuals show better memory for planned actions [15]. A second important factor is social cognition, which is frequently deployed in SM tasks [16] but is deeply altered in ASD [1]. Finally, ASD is characterized by extreme heterogeneity in both intellectual functioning [17] and clinical features [18], which in turn may impact SM to various degrees.

According to the task design, SM performance may, hence, be hard to assess independently of these factors, as these may exert excessive cognitive demands per se in ASD. Identifying shared and independent factors between SM, memory and social cognition may, therefore, contribute to weigh each of these aspects in the functioning of both neurotypical and autistic individuals.

The primary aim of our study was to systematically review the SM literature in ASD. Furthermore, we discussed how the collected findings may help to explain similarities and differences between SM and other cognitive constructs, such as memory type, encoding effects, social cognition, general intelligence, and clinical factors.

## 2. Materials and Methods

### 2.1. Search Criteria

We conducted a systematic literature search in Web of Knowledge and MEDLINE databases using the following search builder: ((source OR reality) AND monitoring) AND (autis* OR ASD). The included timespan started from the date of inception of the database up to 15 November 2020.

**Figure 1:**
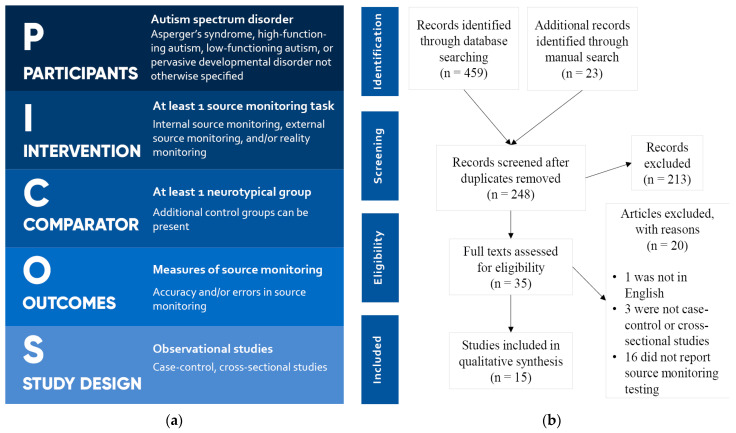
(**a**) PICOS criteria for study selection; (**b**) PRISMA flowchart.

PICOS criteria [19] were used for study selection and are reported in Figure 1a. Articles in languages other than English were excluded. A total of 459 articles were retrieved from the database search, and 23 additional articles were found through manual search. After removal of duplicates, 248 articles were evaluated independently by two authors. After screening, 213 articles were removed, and 35 articles were admitted for full-text evaluation. Of these, 1 was excluded, as it was not in English, 3 were excluded, as they were not case-control or cross-sectional studies, and 16 were excluded, as they did not report SM testing and measures. In the end, 15 articles were included for the systematic review. The process of the literature search and screening is reported in the PRISMA flowchart [20] in Figure 1b.

### 2.2. Quality Assessment

The quality of the included studies was assessed with the Newcastle–Ottawa Scale, considering selection, comparability, and exposure as items [21] as reported in Table A1. Studies were also compared for diagnosis, symptom assessment, demographical factors, and sample numerosity.

### 2.3. Classification of Task Designs

When SM is assessed in experimental settings, the task design is usually structured as follows. Stimuli are first presented to participants together with instructions concerning how to engage with them (for example, to read aloud, to think of a related word, to listen, to observe a picture, to perform a suggested action). The modality of the stimulus presentation and engagement determines the encoding of that information. In the next stage, participants undergo a testing phase where they are asked to make judgments about the source of presented items. In the studies we analyzed, the testing phase often included a memory test. This could be (i) an old–new recognition test (ONRT), where the items of the presentation phase are displayed together with new, distractor items, and participants must recognize which items are old or new; (ii) a recall test, whereby participants are asked to remember which items were presented with (cued recall) or without (free recall) memory support. The test phase may occur immediately after the presentation phase, thus attempting to provide a measure of online SM, or following a planned delay, thus considering the effect of memory. The latter design is more appropriate for the measure of source memory.

The tasks employed in the reviewed studies are described in Table 1.

### 2.4. Data Extraction and Outcome Measures

The following data concerning participants’ clinical and demographical characteristics were extracted from the included studies: diagnosis of cases, definition of controls, number of participants, percentage of males, mean age, intelligence measures and mean values, and additional clinical scores. A description of the SM task, SM type, stimulus modality, involvement of social cognition, and measures of encoding effects were also extracted. For the primary aim, SM performance scores were extracted and qualitatively evaluated. As secondary outcome measures, recognition and recall scores were considered. Definitions for these scores are reported in Table A2. Due to the heterogeneity of the measures used in the included studies, it was not possible to conduct a metanalysis.

## 3. Results

Aims and findings of the reviewed studies are reported in Table 2.

### 3.1. Primary Aim—Source Monitoring Scores

All 15 studies investigated SM. In most of the RM tasks, participants had to discriminate whether an action was performed by either themselves or someone else. For ISM, performance and imagination were compared. ESM mostly relied on visual stimuli, additionally employing auditory stimuli in fewer occasions.

SM accuracy was measured in 14 studies either as the number of sources correctly identified, in proportion to the total number of items presented, or as the proportion of correct responses among correctly identified old items (see Table A2). Overall, seven studies found reduced SM accuracy in ASD [3,13,22,26,28,29]. Bowler and colleagues [27] found that ASD participants had lower SM accuracy when recalling sources without memory support, but when source options were provided, their performance was comparable to controls.

Errors in SM were only reported in three studies, two of which reported a higher number of SM errors in ASD [24,25]. However, Maras and colleagues [25] found that RM was impaired only when free recall was involved, similar to what Bowler and colleagues reported for accuracy in ISM and ESM.

### 3.2. Memory—Old/New Recognition Scores

Performance in ONRT has been investigated by 11 studies. Almost all studies reported no differences in ONRT performance between ASD and control groups, with the exception of Bowler and colleagues, who found lower ONRT signal detection in ASD [27].

### 3.3. Memory—Recall Scores

Among the six studies investigating recall, four found no difference in measures of recall accuracy between ASD and controls [14,23,24,25]. O’Shea and colleagues [28] found that recall accuracy was lower in ASD only for free recall, but not for cued recall. Yamamoto and Masumoto [29] also found decreased free recall accuracy in ASD.

Concerning errors in recall, Maras and colleagues [25] found increased errors in free recall, and were the only study reporting cued recall deficits in ASD.

### 3.4. Encoding Effects

Almost all studies (12 out of 15) included an observation of encoding effects. Eight studies reported no significant between-group differences in enactment effect [3,14,15,22,25,27,29,31], while two studies reported that the enactment effect was absent in both groups [26,30]. Zalla and colleagues [23] found that the enactment effect was intact in SM and ONRT tasks, but impaired in the free recall task. Russell and Jarrold [13] found reduced enactment and an increased observer effect in ASD, in comparison with both neurotypicals and individuals with learning difficulties.

The only study investigating intention superiority effect found it preserved in ASD [15]. Cooper and colleagues [3] found what they called a generation effect: both groups performed better in SM with words that they mentally generated from a hint than with words that they simply read. Finally, Hala and colleagues [26] found that both groups performed better with words that they had only imagined than with words that they pronounced aloud.

### 3.5. Social Cognition

Social cognition was implicated to varying degrees across tasks. No social interaction was requested when ISM alone was measured [15] or when participants only engaged with the computer [32]. Social context was minimal when participants listened to recorded voices [27] or watched people on video [14,23,28]. In nine studies, participants directly observed or listened to the experimenter [3,13,14,22,24,26,29,30,31]. In one study, participants participated in a first aid scenario with the experimenter, thus entailing a higher degree of social interaction, simulating a real-life situation with emotional pressure [25].

Only two studies tried to account for the influence of social cognition on SM. O’Shea and colleagues [28] found that source variables associated with social context—such as human faces or objects close to faces—were related to significantly impaired SM accuracy in ASD, while no difference with controls was found for other source variables, such as furniture. Lind and Bowler [22] assessed theory of mind (ToM, i.e., the ability to understand others’ mental states) [33,34] with an unexpected-contents false-belief task, finding a significantly lower performance in ASD. However, after controlling for the effect of verbal mental age (VMA), the size of the correlation between ToM and SM was not significantly different between ASD and controls.

### 3.6. General Intelligence and Clinical Factors

Measures used to quantify general intelligence are reported in Table 3. All the studies accounted for the influence of intelligence quotient (IQ) and/or VMA, either by matching groups upon enrolment or through additional analyses. In eight studies, ASD IQ mean values were > 80 [13,14,15,23,25,27,28,29]. In two studies, ASD participants had borderline IQ values of 70–80 [3,31]. Five studies involved ASD with intellectual disability (ID) [22,24,26,30,32]. One study [26] found that age and VMA had a significant correlation with RM and ESM in ASD, but not in controls. In another study [22], VMA correlated with both SM and ToM scores in ASD. O’Shea and colleagues [28] adopted VMA as a covariate due to its high correlation with performance in all the cognitive tests that were conducted.

Diagnoses were made according to ICD-10, DSM-IV, or DSM-5 guidelines. Across studies, various ASD diagnoses were indicated for inclusion, including Asperger’s syndrome, high functioning autism, ASD, autism, and pervasive developmental disorder not otherwise specified. Only seven studies assessed ASD core symptoms. The used questionnaires were heterogeneous, Autism Quotient being the most frequently adopted [3,25,29].

Concerning the age of participants, nine studies enrolled children or adolescents, five studies involved adults, and only one study examined both as separate groups.

## 4. Discussion

**Figure 2:**
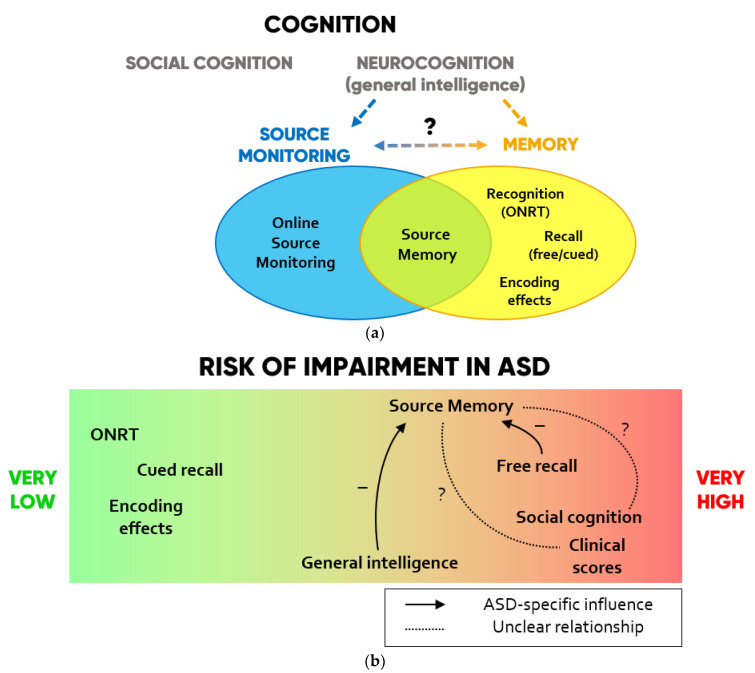
(**a**) Scheme of the cognitive domains investigated in SM studies in ASD. (**b**) Degrees to which each domain is impaired in ASD and hypotheses on ASD-specific influences that each function may exert on SM according to the collected findings.

A conceptual summary of the cognitive resources involved in SM and their preserved or impaired functioning according to the collected findings are displayed in Figure 2.

### 4.1. Source Monitoring Performance and Errors

Even though no consensus can be drawn concerning SM abilities in ASD, the fact that 60% of the studies reported SM impairments must be acknowledged and prompts further investigation.

Only three studies reported SM errors, finding them increased in two instances. Hill and Russell [24] noted that ASD participants had a higher tendency to incorrectly attribute new items to themselves (internalizing errors: RM_int_) when they were asked to recognize whether an action was carried out by themselves, by the experimenter, or was new. In this case, the error concerns both old/new recognition and RM, since new items constitute an external source. Instead, Maras and colleagues [25] reported that when interviewed after a first aid simulation, ASD individuals attributed a higher proportion of their own actions to the experimenter (externalizing errors: RM_ext_) when asked to freely recall events but were no different from controls when they received memory support with guiding questions. No difference in RM_int_ was found.

Such evidence points out that memory, encoding effects, social cognition, general intelligence, and experimental limitations may be crucial to interpret the contradicting findings on SM in ASD.

### 4.2. The Role of Memory in Source Monitoring Tasks

Only 1 out of 11 studies found decreased ONRT performance [27], suggesting that recognition memory is preserved in ASD. This is coherent with the extensive literature reporting intact recognition performance in ASD without ID, while few studies have been carried out in ASD with ID, with mixed results [11].

In the studies we considered, ASD individuals showed difficulties in tasks requiring free but not cued recall [23,27,28,29], with one exception reporting intact free recall [14] and one reporting increased errors in both cued and free recall [25]. In the literature, findings on free recall in ASD are mixed, whereas cued recall seems generally preserved (see review from Boucher and colleagues [11]). This is coherent with the “task support hypothesis” advanced by Bowler and colleagues [35] stating that ASD difficulties in spontaneous memory retrieval can be overcome by task support.

The role of memory in SM tasks is difficult to assess, as the distinction between proper SM and source memory seems to be blurred in the literature. In her definition of SM, Mitchell [7] differentiates online SM as relating to a current event and source memory as concerning events from the past. However, as stated by Johnson and colleagues [8], memory is always involved in SM to a certain degree, and the reviewed tasks entailed the passage of a certain amount of time from the presentation to the test phase with the only exception being the “moving shapes” task. In ASD-related studies, SM has been considered due to its relationship with memory: for instance, Boucher and colleagues [11] classified source memory as a special type of cued memory task that takes place after a recognition test. This view hardly aligns with our findings, as the three studies investigating both SM and cued recall found opposite performances in ASD (when SM was preserved cued recall was impaired and vice versa). This suggests that an excessive focus on this specific memory type may overshadow the dimension of self–other distinction, which is crucial for SM and has been found blurred in ASD-related disorders, such as schizophrenia [36,37].

### 4.3. Encoding Effects

In line with the study from Grainger and colleagues [14], no significant differences in enactment effect were found, except in one study [13] reporting reduced enactment and increased observer effect in ASD. A consistently preserved enactment effect in SM tasks—even when SM is impaired—suggests that action-based self-related encoding in ASD is not responsible for SM deficits [3,22,29].

Considering that self-referential processes have been hypothesized to be atypical in ASD [38], Cooper and colleagues [3] have suggested that future research on SM should distinguish enactment effect, which is related to physical actions, from the self-reference effect, which predicts increased memory for information that is related to the self in a psychological sense [12] and might be altered in ASD. A recent study did not support this hypothesis, finding no evidence of impaired self-reference in ASD [39].

Among the included studies, Grainger and colleagues [15] were the only group investigating the intention superiority effect in ASD. Their study shows that both enactment and intention superiority are preserved in ASD and are significantly correlated with each other, suggesting that they might rely on the same processes, such as motor encoding [40].

### 4.4. Social Cognition

The two studies that directly assessed the influence of social context on SM performance apparently showed opposite trends, but the tasks involved different types of SM. Lind and Bowler [22] observed that ToM correlated with RM performance in neurotypical but not in ASD children after correcting for VMA. O’Shea and colleagues [28] investigated ESM, where accuracy was reduced for two out of the seven source types: the face of the reader and the folder that the reader was holding close to her face. As no other social sources were included as control variables, it is not possible to conclude whether the performance deficit was related to the social context in general, or to the specific difficulty in face processing that is well known in ASD [41,42,43].

### 4.5. Effects of General Intelligence

Findings of preserved or impaired SM were equally distributed between studies with participants with or without ID. However, when the association of IQ/VMA with SM was measured, findings of positive correlations were consistent in ASD. This was not always true for the control groups [22,26,28], suggesting that, unlike neurotypicals, ASD individuals may need to recruit additional cognitive resources to make up for specific deficits in the SM domain deputed to the self–other distinction.

### 4.6. Limitations

Our review has some limitations linked to the heterogeneity of SM evaluation and of ASD characterization in the included studies. The lack of reporting of SM errors in ASD studies is in stark contrast with the practice in schizophrenia studies, where RM_int_ and RM_ext_ are often measured separately, as they may be the results of different neurocognitive processes. SM errors might have been more informative of the source attribution style in ASD than measures of accuracy, possibly leading to further insight that the current literature is lacking. Moreover, different SM accuracy measures were used across studies, possibly affecting the comparability of results. Additionally, some memory components that might have influenced SM performance, such as depth of processing, have not been accounted for.

While a measure for general intelligence was always reported, several other factors were less consistent and reduced the comparability of the reviewed studies. Only four tasks were used in more than one study, leading to high heterogeneity in source types, encoding modalities, and the degree of social context. Designs were also influenced by the fact that one third of the studies primarily focused on cognitive constructs other than SM, such as memory and encoding effects. Only two studies included more than 25 ASD subjects, and age groups and ASD subtypes largely differed between studies. Finally, a characterization of the core ASD symptomatology with standardized questionnaires was rarely provided. The absence of shared evaluation tools in SM mirrors the general fragmentation seen in ASD clinical outcome measures [44,45] and may have contributed to the lack of consensus emerged in the present review.

### 4.7. Toward a Framework for Source Monitoring in Autism

A research framework on SM as a construct of primary interest in ASD is still lacking. The findings collected in present work allow to draft initial guidelines to assist future trajectories and study designs. First, 60% of the studies found altered SM in at least one of its forms, a percentage second only to free recall. Second, implementing online SM tasks may allow to disjoin the self–other distinction and memory components more efficiently. Third, studies aimed at a pure study of SM ability need to use tasks that do not tax working memory differently for ASD and neurotypical populations: to avoid free recall tasks would highly reduce the bias linked to this specific deficit in ASD. Fourth, SM measures should be independent of content memory: to address this issue, sub-scores should be computed whenever different stimulus modalities are considered. Fifth, ONRT and encoding effects are generally unimpaired in ASD, with a negligible relation to SM. Sixth, general intelligence impacted SM to a much higher degree than memory-specific measures, and thus it is more than reasonable to account for it in future studies. Seventh, given that impaired social cognition is a core feature of ASD, SM tasks should differentiate social-related tasks (where the source must be distinguished between people) from social-unrelated tasks, minimizing social interaction during both presentation and testing phases. Lastly, to test for ASD symptoms and their association to SM would improve not only the quality and reliability of the SM findings, but also their applicability within diagnostic and treatment contexts, possibly providing precious advancements on both neurocognitive and clinical grounds [4,46].

## 5. Conclusions

Of the 15 reviewed studies, 9 reported altered SM scores in ASD. While recognition memory and encoding effects were generally preserved, the relationship of memory, social cognition, general intelligence, and clinical factors with SM performance need to be elucidated by future studies. SM in ASD still demands thorough investigation, which will greatly benefit from the consolidation of the experimental settings and outcome measures.

## Figures and Tables

**Table 1:** Task descriptions.

Table 2009	Task Description	Studies
Reality Monitoring		
Card naming	Picture cards are presented with the instruction to be named by either the participant or the experimenter. Participants must then recall who named each item.	Lind and Bowler (2009) [22]
Observe or perform	Actions are shown in a video clip for the participant to either observe or perform the action simultaneously. Participants must then recall whether they had observed or performed them.	Grainger et al. (2014) [14]; Zalla et al. (2010) [23]
Object pairs	Pairs of objects are presented for the participant or the experimenter to perform a certain action. Participants must then recall who performed each action.	Hill and Russell (2002) [24]
First aid scenario	The participant and the experimenter perform some actions in a first aid scenario. Participants must then recall who performed each action.	Maras et al. (2013) [25]
Internal Source Monitoring		
Say or think	Words are presented for the participant to either think or say them out loud. Participants must then recall whether they had thought or said them.	Hala et al. (2005) [26];
Plan or perform	Action phrases are presented for the participant to either read out loud, plan to perform later, or perform now. Participants must then recall whether they had read, planned, or performed them.	Grainger et al. (2017) [15]
Word thinking	Words are presented for participants to either think of a word of similar meaning, rhyming, or longer, or to think of a related action. Participants must then recall what they had done with each word.	Bowler et al. (2004) [27]
External Source Monitoring		
Word presentation	Words are presented either at the top or bottom of a screen, or in a male or female voice. Participants must then recall how they were presented.	Bowler et al. (2004) [27]
Human vs. computer players	Picture cards are placed in cardholders of different colors. The participant, experimenter or computer moves the cards to the central board. Participants must then place each card in the original cardholder	Russell and Jarrold 1999 [13]
Short stories	Short stories read by different actors are shown to participants on video tape. The tapes are characterized by different details. Participants must then recognize the correct details for each video tape.	O’Shea et al. (2005) [28]
Reality and External Source Monitoring		
Listen, observe or perform	Action phrases are presented for the participant to either listen only, to observe the experimenter perform the action, or to perform it. Participants must then recall whether they had listened, observed or performed each action.	Yamamoto and Masumoto (2018) [29]
Red and blue blocks	Either the participant and the experimenter, or two experimenters, hold a red and a blue block. Each presented word is said out loud either by the person holding the red or by the one holding the blue block. Participants must then recall who said each word.	Farrant et al. (1998) [30]; Hala et al. (2005) [26]
Moving shapes	When the mouse is moved, a target shape moves in accordance on a screen, while additional distractor shapes move randomly over increasing levels of difficulty. Either the participant or the experimenter moves the mouse. Participants must then identify which is the shape controlled by the mouse. Therefore, they must try to correctly attribute the source of movement to: (i) either themselves or the computer (ii) either the experimenter or the computer.	Grainger et al. (2014) [14] Williams and Happé (2009) [31]; Russell and Hill (2001) [32]
All Types of Source Monitoring		
Read or imagine	Word pairs are presented to be read by either the participant or the experimenter. Word pairs are either written in full or with the second word being only suggested. Participants must then recall who read each word pair and how were they written.	Cooper et al. (2016) [3]
Human vs. doll players	Picture cards are distributed between participant, experimenter, and two dolls, each controlled by either the participant or the experimenter. In turns, each player lays a card for themselves or their doll partner. Participants must then return each card to the original owner.	Russell and Jarrold (1999) [13]; Williams and Happé (2009) [31]

**Table 2:** Study aims and results.

Study	Primary Aim (ASD vs. CTRL)	Secondary Aims (ASD vs. CTRL)	Stimulus Modality	SM Type	Social Cog.	Performance (ASD vs. CTRL)			
						SM Scores	ONRT Scores	Recall Scores	Encoding Effects
Farrant et al. (1998) [30]	SM <		P, A	RM	yes	SM-A =	ONRT-A = ONRT-E =		No enactment =
Russell and Jarrold (1999) [13]	SM < in human vs. doll	SM = in human vs. computer	P, V	all	yes	SM-A <			Enactment <
						SM-A ESM =			Observer >
Russell and Hill (2001) [32]	SM <		P, V	RM	no	SM-A =			
Hill and Russell (2002) [24]	SM <	ONRT and recall =	P, V	RM	yes	SM-A =	ONRT-A =	REC-A cued =	
						RM_int_ < NT, = MLD			
Bowler et al. (2004) [27]	SM free < SM cued =	Enactment =	I, V, A	ISM, ESM	no	SM-A free <	ONRT-SD <		Enactment =
						SM-A cued =			
Hala et al. (2005) [26]	RM < but ONRT =	SM < when self is agent (RM, ISM)	P, I, V	all	yes	SM-A <	ONRT-A = ONRT-E =		No enactment = I > P =
O’Shea et al. (2005) [28]	SM <	Source variables recognition ≠	V	ESM	yes	SM-A <	ONRT-E =	REC-A free <	
						SM-E =		REC-A cued =	
Lind and Bowler (2009) [22]	SM < but ONRT =	Enactment =	P, A	RM	yes	SM-A <	ONRT-SD =		Enactment =
Williams and Happé (2009) [31]	Self-reference <	Observer > self-reference	P, V	all	yes	SM-A =			Enactment =
Zalla et al. (2010) [23]	Enactment =		P, V	RM	no	SM-A =	ONRT-SD =	REC-A free =	Enactment < recall
									Enactment = SM, ONRT
Maras et al. (2013) [25]	Enactment < in free SM = in cued SM	SM-E < in free SM, = in cued SM	P, V	RM	yes	RM_int_ =		REC-A =	Enactment =
						RM_ext_ free <		REC-E free <	
						RM_ext_ cued =		REC-E cued <	
Grainger et al. (2014) [14]	Action monitoring =	Enactment =	P, V	RM	yes	SM-A =	ONRT-SD =	REC-A free =	Enactment =
Cooper et al. (2016) [3]	Enactment <	SM < for self-other sources	P, I, A	all	yes	SM-A<	ONRT-SD =		Enactment = Generation =
Grainger et al. (2017) [15]	Intention superiority <	Action monitoring =	P, I	ISM	no	SM-A =	ONRT-SD =		Enactment = Intention =
Yamamoto and Masumoto (2018) [29]	Self-reference <		P, V, A	RM, ESM	yes	SM-A<	ONRT-A= ONRT-E=	REC-A free<	Enactment=

For primary aim, secondary aims, and performance, < (red color) indicates worse performance of ASD group compared to CTRL group, while = (green color) indicates the absence of significant difference in performance between groups. A = auditory; ASD = autism spectrum disorder; CTRL = controls; ESM = external source monitoring; I = imagined; ISM = internal source monitoring; ONRT = old-new recognition test; ONRT-A = ONRT accuracy; ONRT-E = ONRT errors; ONRT-SD = ONRT signal detection; P = performed; REC-A = recall accuracy; REC-E = recall errors; RM = reality monitoring; RM_int_ = RM internalizing errors; RM_ext_ = RM externalizing errors; SM = source monitoring; SM-A = SM accuracy; SM-E = SM errors; Social cog. = social cognition; V = visual.

**Table 3:** Study characteristics and demographics.

Study	ASD Type	CTRL	N Subjects		% Males		Age Mean		Intelligence Measure	Intelligence Mean		Clinical Scores
			ASD	CTRL	ASD	CTRL	ASD	CTRL		ASD	CTRL	
Farrant et al. (1998) [30]	AUT	NT MMR	15	15 15	93.3	86.7 86.7	12.7	7.6 12.9	BPVS (VMA)	7.8	7.7 7.6	
Russell and Jarrold (1999) [13]									BPVS (IQ)			DSM III-R
*- Task 1*	AS, AUT	NT MLD	22	22 22			13.2	6.8 11.3		85.6	85.4 86.6	
*- Task 2*	AS, AUT	NT	19	19			13.8	7.3		88	87.9	
		MLD		19				11.9			87.5	
Russell and Hill (2001) [32]	AS, AUT	NT MLD	28	28 28	75	57.1 32.1	10.1	6.09 10.06	BPVS (VMA)	6.1	6.1 6	
Hill and Russell (2002) [24]	AUT	NT MLD	20	20 20	75	60 50	9.8	6 9.6	BPVS (VMA)	5.9	5.9 6	
Bowler et al. (2004) [27]	AS AS	NT NT	16 16	16 16	93.8 100		13.5 34.5	13.4 33.4	BPVS (IQ) WAIS-R (IQ)	100.8 100.2	94.6 97	
Hala et al. (2005) [26]	AUT	NT	13	13	84.6	84.6	8	6	PPVT (VMA)	6	6	
O’Shea et al. (2005) [28]	AUT, PDD-NOS	NT	21	21	81	42.9	10.9	10.6	WISC-III (IQ)	94.7	101.8	ADOS
Lind and Bowler (2009) [22]									BPVS (VMA)			
*- Task 1*	AS, AUT	NT, ID	53	50	84.9	70	9.3	9.09		6.7	6.5	
*- Task 2*	AS, AUT	NT, ID	73	55	82.2	67.3	10.1	8.6		6.6	6.1	
Williams and Happé (2009) [31]									WISC-III, BPVS (IQ)			
*- Task 1*	AS, AUT, PDD-NOS	ID	16	16			13.4	13		72	69.9	
*- Task 2*	ASD	ID	16	16			12.4	12.2		73.5	67.4	
Zalla et al. (2010) [23]	AS	NT	18	18	83.3	77.8	26.2	27.7	WAIS-III (IQ)	107.4	106.7	ADI-R
Maras et al. (2013) [25]	AS, AUT	NT	18	18	88.9	83.3	41.1	45.5	WAIS-R (IQ)	109.8	110.7	ADOS, AQ
Grainger et al. (2014) [14]	AS, AUT	NT	17	17			29.1	29.4	WASI (IQ)	114.5	113.6	
Cooper et al. (2016) [3]	AS, HFA	NT	24	24	45.8	45.8	31.4	30.5	RM-sf (IQ)	75.4	70.8	AQ
Grainger et al. (2017) [15]	AS, AUT	NT	22	20	86.4	100	13.4	13.2	WASI (IQ)	106.7	109.5	SRS
Yamamoto and Masumoto (2018) [29]	ASD	NT	14	16	57.1	43.8	30.5	27.9	WISC-III (IQ)	103.6	106.4	AQ

Groups: AS = Asperger’s syndrome; ASD = autism spectrum disorder; AUT = autism; CTRL = controls; HFA = high-functioning autism; ID = intellectual disability; MLD = moderate learning difficulties; MMR = mild mental retardation; NT = neurotypical; PDD-NOS = pervasive developmental disorder not otherwise specified. Scales: ADI-R = Autism Diagnostic Interview-Revised; ADOS = Autism Diagnostic Observation Schedule; AQ = Autism Quotient; BPVS = British Picture Vocabulary Scale; DSM III-R = Diagnostic and Statistical Manual of Mental Disorders-Third Edition-Revised; IQ = Intelligence Quotient; PPVT = Peabody Picture Vocabulary Test; RM-sf = Raven’s Matrices-short form; SRS.

## Appendix A

**Table A1:** Newcastle–Ottawa Scale for assessment of study quality.

Study	Selection (★★★★)	Comparability (★★)	Exposure (★★★)	Total Score (9)
Farrant et al. (1998) [30]	★ ★	★ ★	★ ★ ★	7
Russell and Jarrold (1999) [13]	★ ★	★	★ ★ ★	6
Russell and Hill (2001) [32]	★ ★ ★	★	★ ★ ★	7
Hill and Russell (2002) [24]	★ ★ ★ ★	★	★ ★ ★	8
Bowler et al. (2004) [27]	★ ★ ★	★ ★	★ ★ ★	8
Hala et al. (2005) [26]	★ ★ ★	★	★ ★	6
O’Shea et al. (2005) [28]	★ ★ ★ ★	★ ★	★ ★ ★	9
Lind and Bowler (2009) [22]	★ ★ ★	★ ★	★ ★ ★	8
Williams and Happé (2009) [31]		★ ★	★ ★ ★	5
Zalla et al. (2010) [23]	★ ★ ★	★ ★	★ ★ ★	8
Maras et al. (2013) [25]	★ ★ ★ ★	★	★ ★ ★	8
Grainger et al. (2014) [14]	★ ★ ★ ★	★ ★	★ ★ ★	9
Cooper et al. (2016) [3]	★ ★ ★ ★	★ ★	★ ★ ★	9
Grainger et al. (2017) [15]	★ ★	★ ★	★ ★ ★	7
Yamamoto and Masumoto (2018) [29]	★ ★ ★ ★		★ ★ ★	7
Mean values				7.5

Scores in each category have been indicated as stars (★). Maximum scores for each category are indicated between brackets.

**Table A2:** Definitions of source monitoring and memory scores.

Outcome Measure	Score	Definition
Source Monitoring		
Errors (SM-E)	External Internal	Incorrectly identified external item sources Incorrectly identified internal item sources
	Reality internalizing (RM_int_)	External item sources incorrectly identified as internal
	Reality externalizing (RM_ext_)	Internal item sources incorrectly identified as external
Accuracy (SM-A)	Source score	Correctly identified sources/N° old items correctly recognized as old
	Source proportion	Correctly identified sources/total N° of items presented
	Source number	Correctly identified sources
Memory—Old/New Recognition (ONRT)		
Errors (ONRT-E)	False alarm rate (FA)	Proportion of new items incorrectly recognized as old
	Miss rate (MISS)	Proportion of old items incorrectly recognized as new
	FA+MISS	FA errors + MISS errors
Accuracy (ONRT-A)	Hit rate (H)	Proportion of old items correctly recognized as old
	Recognition number	N° of old items correctly recognized as old
Signal detection (ONRT-SD)	Corrected hit rate	H–FA
	Recognition performance	z(H)–z(FA) ^1^
	Item discrimination	1/2 + [(H − FA)(1 + H − FA)]/[(4H)(1 − FA)]
Memory—Free or cued recall		
Errors (REC-E)	Omission errors	N° of items incorrectly not recalled
	Commission errors	N° of items incorrectly recalled
Accuracy (REC-A)	Recall number	N° of items correctly recalled
	Recall proportion	Proportion of items correctly recalled

^1^ z = Z-score, the number of standard deviations the given value differs from the mean.
